# One origin for metallo-β-lactamase activity, or two? An investigation assessing a diverse set of reconstructed ancestral sequences based on a sample of phylogenetic trees

**DOI:** 10.1007/s00239-014-9639-7

**Published:** 2014-09-04

**Authors:** Rosanna G. Alderson, Daniel Barker, John B. O. Mitchell

**Affiliations:** 1 Biomedical Sciences Research Complex and EaStCHEM School of Chemistry, Purdie Building, University of St Andrews, North Haugh, St Andrews, KY16 9ST Scotland, UK; 2 Sir Harold Mitchell Building, School of Biology, University of St Andrews, St Andrews, KY16 9TH Scotland, UK

**Keywords:** Metallo-β-lactamase, Antibiotic resistance, Phylogenetics, Most recent common ancestor, Ancestral sequence reconstruction, Exaptation

## Abstract

Bacteria use metallo-β-lactamase enzymes to hydrolyse lactam rings found in many antibiotics, rendering them ineffective. Metallo-β-lactamase activity is thought to be polyphyletic, having arisen on more than one occasion within a single functionally diverse homologous superfamily. Since discovery of multiple origins of enzymatic activity conferring antibiotic resistance has broad implications for the continued clinical use of antibiotics, we test the hypothesis of polyphyly further; if lactamase function has arisen twice independently, the most recent common ancestor (MRCA) is not expected to possess lactam-hydrolysing activity. Two major problems present themselves. Firstly, even with a perfectly known phylogeny, ancestral sequence reconstruction is error prone. Secondly, the phylogeny is not known, and in fact reconstructing a single, unambiguous phylogeny for the superfamily has proven impossible. To obtain a more statistical view of the strength of evidence for or against MRCA lactamase function, we reconstructed a sample of 98 MRCAs of the metallo-β-lactamases, each based on a different tree in a bootstrap sample of reconstructed phylogenies. InterPro sequence signatures and homology modelling were then used to assess our sample of MRCAs for lactamase functionality. Only 5 % of these models conform to our criteria for metallo-β-lactamase functionality, suggesting that the ancestor was unlikely to have been a metallo-β-lactamase. On the other hand, given that ancestral proteins may have had metallo-β-lactamase functionality with variation in sequence and structural properties compared with extant enzymes, our criteria are conservative, estimating a lower bound of evidence for metallo-β-lactamase functionality but not an upper bound.

**Electronic supplementary material:**

The online version of this article (doi:10.1007/s00239-014-9639-7) contains supplementary material, which is available to authorized users.

## Background

The ability to hydrolyse a lactam molecule is necessary for the survival of antibiotic resistant bacteria. Two broad classes of mechanisms to hydrolyse lactam rings have evolved, one using a serine residue and the other using zinc to activate water in nucleophilic attack. Enzymes using the latter type of mechanism are classified as metallo-β-lactamases and are able to hydrolyse a wide range of substrates (Bebrone 2007), conferring resistance to a broad range of antibiotics. Resistance to antibiotics predates their use in medicine (D’Costa et al. 2011; Coulson 1985), as metallo-β-lactamase function is believed to have first arisen more than two billion years ago (Hall et al. 2004), although it is the intensive use of antibiotics by the human population that has accelerated the recent well-publicised emergence of resistant strains (Oelschlaeger 2008). A pressing concern for our understanding of the evolution of resistance, and for our understanding of evolutionary processes, is whether this enzyme function has evolved once only, or more than once through independent origins of this function within the metallo-β-lactamase superfamily.

The metallo-β-lactamase superfamily (CATH 3.60.15.10) (Sillitoe et al. 2013) consists of a diverse set of enzymes including the A-type flavoproteins, glyoxalase IIs and the metallo-β-lactamases. These are clustered by the protein structure–function phylogeny suite FunTree (Furnham et al. 2012a, b) into two ‘Structurally Similar Groups’: ‘SSG1’, including the metallo-β-lactamases; and a second group, ‘SSG2’, structurally distinct from the first group and including the RNase Z enzymes. The metallo-β-lactamases consist of three subclasses: B1, B2 and B3 (Galleni et al. 2001). The B1 and B2 subclasses are more closely related to each other than to the B3 group (Hall et al. 2003). The B1/B2 and B3 subclasses of metallo-β-lactamases share common mechanistic features, in which zinc activates a water molecule which then carries out nucleophilic attack on the carbonyl carbon of the lactam ring, resulting in hydrolysis of the amide bond. However, stabilisation of the transition state is achieved by different residues in the B1/B2 and B3 subclasses (Wang et al. 1999b; Spencer et al. 2005; Ullah et al. 1998; Wang et al. 1999a; Xu et al. 2007). Innovation of function in this superfamily seems to generally depend on changes in transition state stabilising residues within a preserved ancestral active site scaffold that has evolved to accommodate different substrates, as discussed by Aravind (1999) and in a wider context for different enzyme families by Todd et al. (2002) and Anantharaman et al. (2003). In this respect, these subclasses could be thought of as distinct in function, and their classification should reflect this, as argued by Hall and Barlow (2005).

Both Aravind and Hall and Barlow postulated independent origins of the B1/B2 and B3 subclasses (Hall et al. 2003, 2004; Aravind 1999). This was based on differences in sequence, structure and, in the case of work by Hall and Barlow, phylogenetic mapping of antibiotic resistance of extant enzymes which led them to date the origination of B1/B2 activity at one billion years ago and B3 activity two billion years ago (Hall et al. 2003, 2004). Indeed, at the sequence level, the B1/B2 and B3 subclasses appear very different, indicative of a divergence from a common ancestor far back in evolutionary history. Whether these groups constitute products of discrete independent evolutionary functional innovations is difficult to determine. In such cases, analysis of structure can yield extra information. Although structurally alignable, both the B1/B2 and B3 groups have different and discrete structural features, making the inference of evolutionary history based on this structural evidence ambiguous (Wang et al. 1999b).

Independent evolution of the same function, most often using different mechanisms but occasionally using different catalytic machineries for essentially the same mechanism, is well documented for proteins from different non-homologous enzyme families (Gherardini et al. 2007). However, this phenomenon seems rarer within homologous superfamilies, with relatively few examples in the literature (Bruns et al. 1997; Burroughs et al. 2006). Partly this is expected, because of the smaller scope for evolutionary change within a superfamily (as opposed to across all sequences). However, convergence to a similar function is possible even over relatively small evolutionary time scales, given sufficiently strong selective pressures; and members of the same family may be structurally exapted (preapted) to evolve this same novel function. There are examples of independent evolution of function within homologous families of enzymes with significant roles in host-pathogen relationships such as the iron-transporter ferric ion-binding protein found in *Haemophilus influenzae* (Bruns et al. 1997), in plant resistance genes (Ashfield et al. 2004) and in the phosphatidylinositol-phosphodiesterase superfamily, where similar functionality has been achieved by different catalytic mechanisms and includes a member that catalyses the production of sicariid spider venom (Furnham et al. 2012a, b). Examples have also been proposed in which pathogens have evolved proteins with similarities to host homologs via convergent evolutionary mechanisms, increasing virulence (Sikora et al. 2005).

In this work, we ask whether the B1/B2 and B3 subclasses have diverged from some ancestral β-lactamase, or whether the same mechanism of lactam hydrolysis has evolved twice independently, within the same superfamily, from ancestral proteins with no β-lactamase activity. As a first step, an accurate phylogenetic reconstruction for the sequences in the family must be sought. Both our current study and earlier studies demonstrate the difficulty of unambiguously resolving phylogenetic relationships based on extant sequences in this superfamily (e.g. Garau et al. 2005). The use of a maximum-likelihood (ML) tree-building strategy, combined with bootstrapping the multiple alignment to obtain an indication of clade support and a sample of phylogenetic trees over which to perform ancestral reconstructions (e.g. Latysheva et al. 2012), seemed most appropriate in this case. We did not pursue the alternative strategy of obtaining a sample of phylogenetic trees using Bayesian Markov chain Monte Carlo (e.g. Lutzoni et al. 2001). There are several reasons for this—discussed in further detail in the “Discussion” section.

**Fig. 1:**
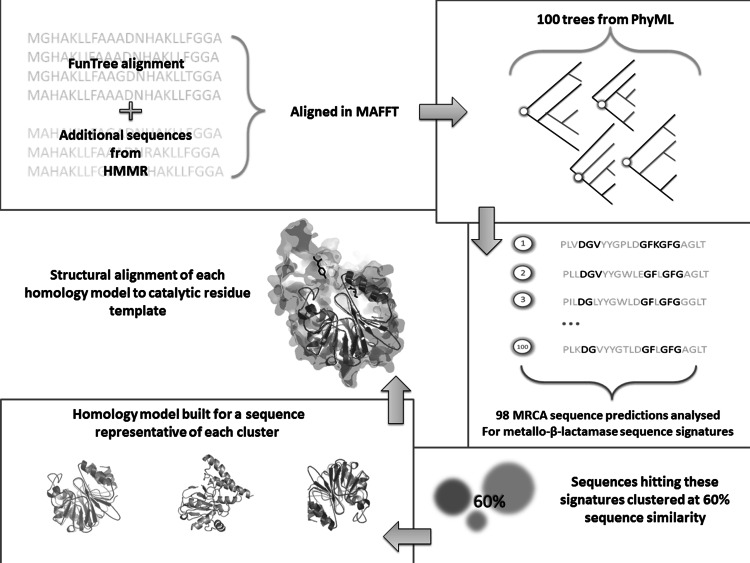
Schematic diagram of the MRCA approach using a bootstrap sample. Additional sequences were aligned to the pre-existing FunTree alignment. This alignment was then used to build a maximum-likelihood tree, with 100 bootstrap replicates. The MRCA sequence was obtained from 98 of the trees in the bootstrap set. The 98 sequences were clustered at 60 % sequence identity. A representative from each cluster was submitted to the homology modelling server PHYRE2. Functional analysis was then carried out on each of these homology models compared to pre-constructed active site templates

For the prediction of ancestral sequences we chose to use GASP, as it is a heuristic, probabilistic approach particularly suitable for gapped alignments. Overall, our approach (Fig. 1) to this question differs from that of previous studies. Firstly, we reconstruct the ancestral sequence for all three subclasses. Secondly, rather than basing this estimate on a single reconstructed phylogenetic tree, we use a sample of 100 trees (Felsenstein 1988; Lutzoni et al. 2001; Pagel et al. 2004; Latysheva et al. 2012). Additionally, we reconstruct the ancestral sequence not at a specific node, but for the most recent common ancestor (MRCA) of the B1, B2 and B3 lactamases (Pagel et al. 2004). The 98 resulting MRCA sequences were then clustered by sequence similarity and analysed using InterPro signatures, homology modelling and structural alignments. This allows us to assess the catalytic properties of the common ancestor without unrealistic assumptions concerning the reliability of a single tree or the precise position of the ancestor on the tree. Three-dimensional (3D) catalytic templates were used to discern lactamase function since sequence signatures and even global structural similarity are not always adequate confirmation of functionality. Here, we follow the lead of others in inferring function by the 3D location of catalytic residues (Meng et al. 2004; Torrance et al. 2005).

Phylogenetics can be used to make informed decisions as to which resistance genes and organisms to study, important for future antibiotic design efforts (Hall 2004). We cannot prepare for all the possible trajectories that evolution could take, but studying past evolutionary patterns and processes can help highlight more likely ones (Bush et al. 1999; Plotkin et al. 2002; Lemey et al. 2007; Palmer and Kishony 2013). In this case, if evolution has independently ‘invented’ the same function more than once in this superfamily, then one might fear that the fold can accommodate the hydrolysis of a wide range of substrates relatively easily, and may be exapted to bind and effectively hydrolyse β-lactam substrates—with worrying consequences for the future development of antibiotic resistance.

## Results

### Phylogenetic tree

**Fig. 2:**
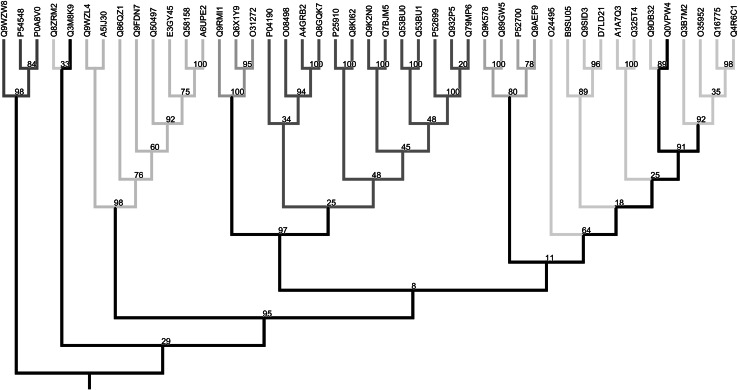
Rooted phylogenetic tree with percentage bootstrap support values. Tips are identified by UniProtKB accession numbers. Enzyme groups are colour coded by function as follows—*red* ribonucleases, *cyan* glyoxalase IIs, *green* A-type flavoproteins, *pale pink* subclass B2 metallo-β-lactamases, *magenta* B1 metallo-β-lactamases, *orange* B3 metallo-β-lactamases, *black* no function assigned. The phylogeny was visualised using Mesquite (Maddison and Maddison 2011)

The low bootstrap support of many nodes in our maximum-likelihood tree illustrates the difficulty of reconstructing relationships between these ancient functional groups (Fig. 2). However, monophyly of each of the groups of functions is strongly supported (each forming a clade with high bootstrap support) apart from a glyoxalase II (UniProt: Q8ZRM2) (The Uniprot Consortium 2013) from *Salmonella typhimurium*, which falls in a small clade basal to the rest of the ingroup. However, the bootstrap support for this placement of Q8ZRM2 is low. We only used 98 out of 100 trees in the bootstrap set for further analysis, since only in these was the ingroup monophyletic (Online Resource 1); hence, our results are conditional on the monophyly of the ingroup (Buschbom and Barker 2006).

### InterProScan prediction and clustering

Forty-four of the 98 MRCA sequence predictions (Online Resources 2 and 3) hit InterPro signature IPR001018 (Zdobnov and Apweiler 2001; Quevillon et al. 2005), known as *Beta*-*lactamase, class*-*B, conserved site*. We used matches to IPR001018 as an initial necessary but not sufficient criterion for having metallo-β-lactamase activity. This was based on results when using functionally characterised extant members of our trees as test cases. We found that, although not all metallo-β lactamases had signature IPR001018, nearly all enzymes with this signature were metallo-β-lactamases. IPR001018 in fact combines two separate ProSite (Sigrist et al. 2013) signatures: PS00743 describes zinc binding and catalytic residues, and is hit by both B1/B2 and B3 sequences; and PS00744 is hit only by subclasses B1/B2.

Clustering of these 44 sequences was performed using CD-HIT (Huang et al. 2010) at a 60 % cutoff level (Online Resource 4). It is likely that at this level, the clusters would still contain members that would fold into a similar 3D structure (Chothia and Lesk 1986), whilst keeping the number of clusters low enough to perform homology modelling on one representative of each cluster.

### Homology modelling of MRCA sequences and alignment to metallo-β-lactamase templates

**Fig. 3:**
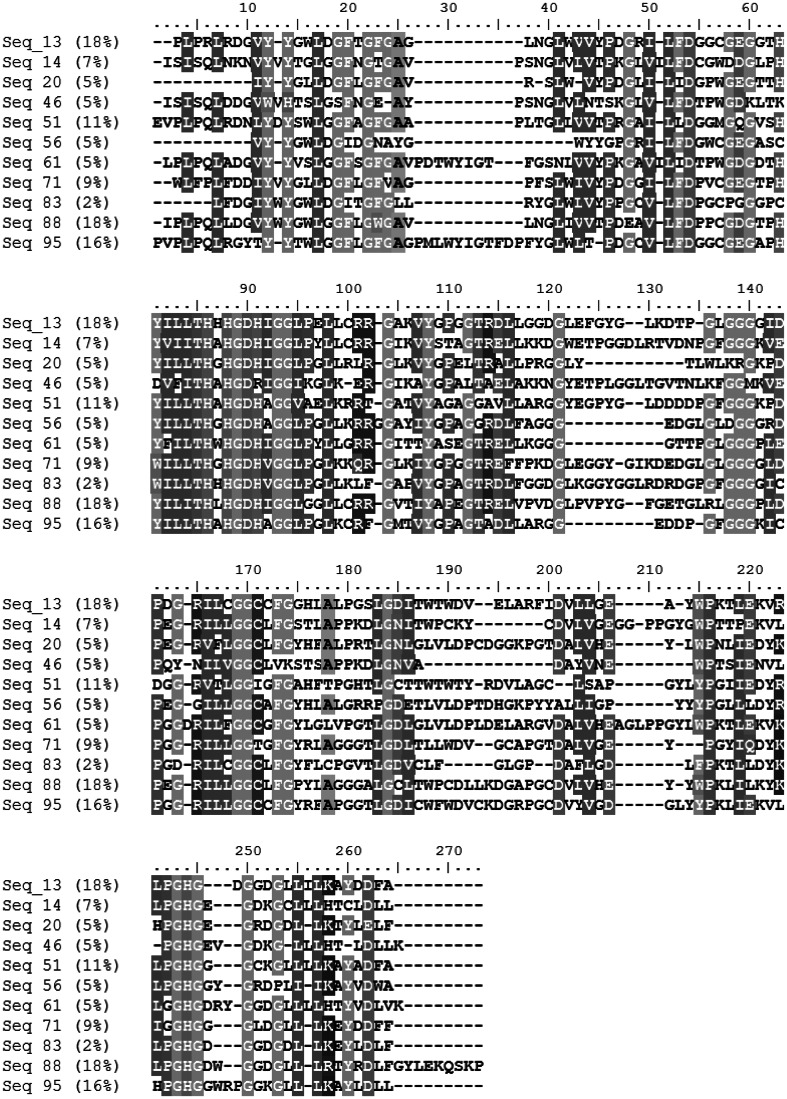
Representative sequences from each of the 11 clusters with weightings. Sequence alignment of each representative from each cluster, aligned with default settings in MAFFT. Each percentage value represents the weight of the cluster from the 44 MRCA sequences with IPR001018 signatures. Columns are coloured at a 70 % similarity threshold. The sequence alignment was visualised in BioEdit (Hall 1999)

**Fig. 4:**
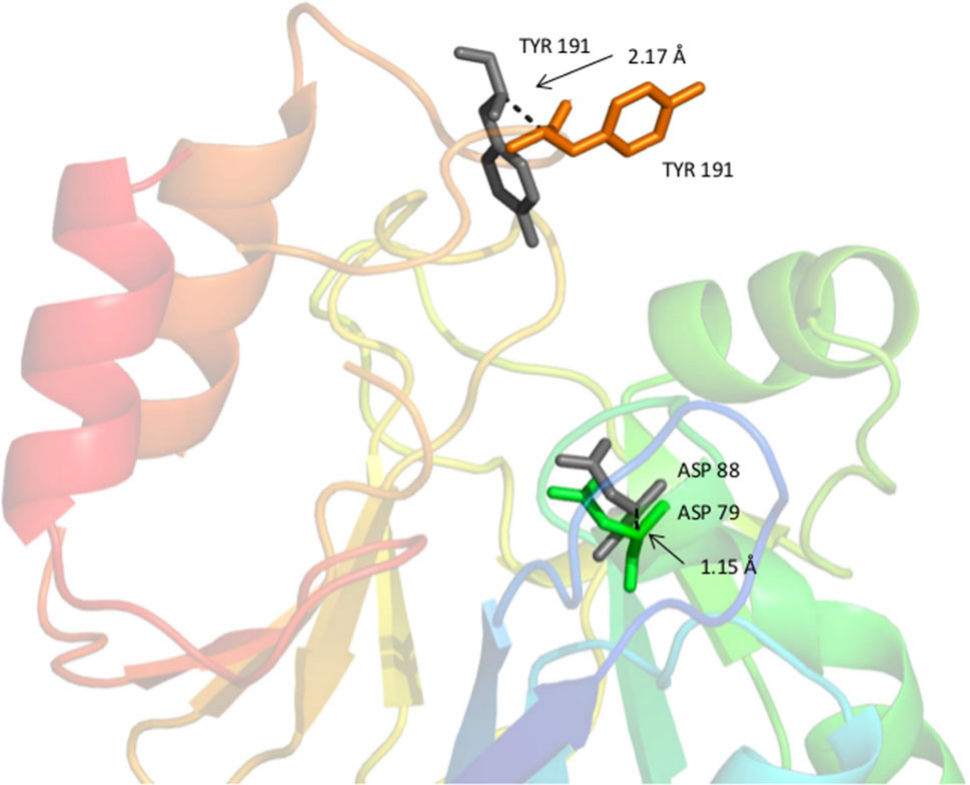
Homology model of an MRCA with possible metallo-β-lactamase functionality. The sequence representative number 51 passed both criteria for being most like a B3 metallo-β-lactamase. The PHYRE2 homology model (cartoon, rainbow) aligned with 1SML with an RMSD of 3.5 angstroms. A zoomed in image of each homology model’s predicted catalytic residues is shown. Homology model predicted residues ASP (*green*) aligned with catalytic ASP residues in the B3 template (*grey*). Homology model predicted residues TYR (*orange*) aligned with catalytic TYR residues in the B3 template (*grey*). Distances are shown in angstroms. Image was generated using Pymol (Schrodinger LLC 2010)

Eleven MRCA sequence representatives (Fig. 3), one from each CD-HIT cluster, were submitted to the PHYRE2 homology modelling server (Kelley and Sternberg 2009). The 11 MRCA homology models were variable in global structural similarity, with some representatives being most similar to metallo-β-lactamases and others being more similar to A-type flavoproteins and even a sec-alkylsulfatase. Typically, one would search for the presence of catalytic machinery using the 3D Jess templates that are searchable via the ProFunc server (Laskowski et al. 2005). However, tests with known PDB (Bernstein et al. 1977) structures indicated that 3D matches occurred for B1 but not for B3 active sites, there being no existing template that is matched by a typical B3 configuration of catalytic residues. Hence, we instead generated our own catalytic templates, as described in the Methods section. Two out of 11 clusters had representative sequences that, when homology modelled and structurally aligned to our metallo-β-lactamase templates, had a residue with the same identity as the template within a 5-angstrom radius. We further filtered these five MRCA models by measuring the distance between key catalytic residues and setting a threshold cutoff based on distance observed between catalytic residues in the corresponding active site template (further described in the Methods section). Only one sequence representative (sequence 51) remained after this step. This sequence was most like a B3 metallo-β-lactamase (Fig. 4) that was a representative of a cluster with five members, and so supported five of the 98 phylogenetic trees in the bootstrap set. It should be noted that sequence 46 was also a very close candidate for having B1 functionality (closest to PDB template 1M2X), but its homology model had a distance just over the ±2.0 Angstrom cutoff between ‘catalytic’ residues as compared to the template (Online Resource 5).

## Discussion

The ambiguity in relationships between functionally distinct groups of enzymes in our maximum-likelihood tree describing the evolution of this superfamily is indicative of the difficulty in resolving these ancient events. This is a problem that has been encountered by others too, for example Garau et al. (2005). Although, just as in our tree, the B1 and B2 subclasses appear more closely related relative to the B3 subclass—in line with the work of Hall and Barlow (Hall et al. 2003, 2004; Hall and Barlow 2005)—the tree proposed by Garau et al. (2005) was not well resolved in the proposed divergence of the B3 metallo-β-lactamases from the glyoxalases.

Although we recognise that unambiguously reconstructing the evolutionary relationships of these groups of functionally related enzymes is a challenge, it is possible to make a plausible range of ‘best guesses’ using a rigorous phylogenetic inference method. Here, we represent phylogenetic uncertainty using a bootstrap sample of phylogenies, which improves on previous phylogenetic work in this area. An alternative strategy might have been to use a Bayesian approach. This would be beneficial from the viewpoint of mathematical interpretability, since the assignment of posterior probabilities to individual trees and nodes provides a clear quantification of our degree of belief in a given node and ancestral sequence. However, these probabilities are conditional on the phylogenetic model applied, the exact multiple alignment used and the priors and, hence, may be difficult to interpret on biological grounds. Bootstrapping with ML phylogeny reconstruction, though still sensitive to the model applied, does not require priors and also gives an indication of the robustness of results in the face of sampling error (Alfaro 2003). In fact, from a practical viewpoint, a bootstrap method of sampling provides inherently greater variability and, therefore, may help avoid drawing conclusions with a higher certainty than is, biologically, warranted. It seemed sensible to choose a method that is not reliant on prior assumptions, since existing data on the family are ambiguous as previously discussed. One attempt to reflect prior ignorance in a Bayesian framework is the use of uniform priors. However, for continuous variables that can take any non-negative value, such as branch length, a uniform prior is not feasible. Yet, a prior distribution with finite parameters (e.g. a geometric distribution specified by its mean) would introduce unwanted subjectivity (Yang and Rannala 2005; Yang 2006, p. 180). Secondly, even if prior knowledge could be assumed for our data, it is well known that setting a prior on one parameter such as topology implies a non-uniform prior on other aspects of the solution, such as clade size (Yang and Rannala 2005; Autzen 2011; Barker 2014). Thirdly, whilst a strong signal within the data reduces the influence of the prior (whatever it is), phylogenetic signal in our multiple alignment is relatively weak, in practice accentuating the difficulty of formulating appropriate priors.

It is well recognised that using a phylogenetic approach to estimate ancestral sequences is more accurate than not, even if the tree is uncertain (Hanson-Smith et al. 2010; Risso et al. 2014). However, there is some debate in the field as to the optimal strategy for ancestral sequence reconstruction. For example, the increasingly popular Bayesian approach (e.g. used in studies by Butzin et al. 2013 and Risso et al. 2013) has been predicted to be more robust in its prediction of the thermostability of ancestral sequence compared to ML (Williams et al. 2006). However, Hall 2006 added further evidence that despite these better predictions of thermostability that Bayesian reconstruction affords, the accuracy of reconstruction for protein sequences is higher with ML. In fact, Hobbs et al. (2012) demonstrated that at least in the case of reconstruction of an ancestral metabolic enzyme 3-isopropylmalate dehydrogenase, Bayesian reconstruction leads to estimates of thermostability that, when compared to ML-based analysis, generated predictions that were kinetically unrealistic.

In this study, we have selected a protein substitution model that suits the data; used bootstrapping to obtain a range of estimates of phylogenetic trees; and constructed various structural models from MRCA predictions from this tree set.

Having generated representative MRCA sequences and structures, the only available in silico means of assigning function to them was by matches to sequence signatures and structural templates. Such matches contain two types of information: firstly that the query protein shares common ancestry with proteins of the known function, and secondly that the query protein contains certain key residues positioned to act as the catalytic machinery. In most bioinformatics and function prediction applications, these two kinds of information add weight to one another. Here, however, we should ideally prise them apart, since homology is a given (at the level of superfamily membership), and it is the presence of viable catalytic machinery that we are trying to discern. The two sequence signatures here are short enough to reflect the presence of short regions containing functionally critical conserved residues; our structural templates describe essential residues necessary, but not necessarily sufficient, for lactamase activity.

It is here that we come back to our fundamental question, did the most recent common ancestor of the B1/B2 and B3 metallo-β-lactamases have lactamase activity? According to our strictest criteria, only five out of 98 of trees in the bootstrap sample give a common ancestor with putative metallo-β-lactamase activity. This may be seen as supporting the existing conclusion that the metallo-β-lactamase fold has evolved lactam hydrolysis on two separate occasions. The active sites of members of this superfamily appear to share a conserved scaffold, with changes in substrate corresponding to changes in identity and locations of key residues stabilising the transition state (Furnham et al. 2012a, b; Holliday et al. 2012; Porter et al. 2004), as is seen in our 3D catalytic site templates. However, our criteria are based on extant metallo-β-lactamases. The full range of metallo-β-lactamases that have ever existed may have a greater diversity of sequence and structural features than are seen in the smaller subset of extant sequences that has been subject to experimental study. For example, a study by Risso et al. (2013) experimentally resurrected Precambrian Class-A β-lactamases and found that they had increased thermal hyperstability and substrate promiscuity compared to modern enzymes. A review discussing enzyme evolution and promiscuity was published by Alderson et al. (2012).

A further, general problem for ancestral sequence reconstructions is the use of a phylogenetic model that considers mutations probabilistically at the granularity of the single residue. This is in common with the great majority of phylogeny reconstruction methods, for example Ashkenazy et al. (2012), Yang et al. (1995) and Menzel et al. (2011). Some allow autocorrelation between neighbouring sites (Yang 1995; Felsenstein and Churchill 1996) or classes of sites that are not necessarily adjacent (Pagel and Meade 2004) or use a Covarion (Concomitantly variable codon) model (Fitch 1971), but still, necessarily, without any consideration of the biological plausibility of mutations persisting. In reality, the consequences of mutations at different positions will interact, and their probability of persisting will depend on selection pressures and population size.

Presumably, at most stages within the phylogeny of the superfamily, its members will have been foldable and will have made a positive contribution to fitness. Nothing in our model imposes a tendency for maintenance of functional ‘usefulness’ (i.e. a positive contribution to fitness) or foldability over evolutionary time. Although it is obviously desirable to model evolution accurately, this is currently impossible due to our near-total lack of knowledge of population sizes, selection pressures and generation times up to two billion years in the past. Despite such simplifications, the ability of phylogeny reconstruction methods to reconstruct phylogenies plausible from a protein-structure point of view is encouraging (Lakner et al. 2011). In common with other studies reconstructing ancestral sequences, we can expect that our in silico evolutionary trajectories are only broadly representative of possible pathways from a MRCA to the extant B1/B2 and B3 metallo-β-lactamases. For example, are the 54 MRCA sequences that did not fit an InterPro signature with a specific function just scrambled estimates of ancestors in which all functional signal had been lost? Or, could these 54 ‘others’ be accurate estimates of ancestors in which the functional signal is unknown? There are numerous extant ‘superfamily proteins’ which have been discovered (Yamamura et al. 2008; Alfredson and Korolik 2007; Shimada et al. 2010), and yet have no function assigned to them and match no functional signature in InterPro.

Without a current method to distinguish functional (enzymatic or not) from non-functional sequences, we have turned our attention to the 44 sequences that hit IPR001018 and, therefore, had the potential, at the sequence level at least, to be a metallo-β-lactamase, according to criteria based on extant sequences of known function. At first glance, the fact that only five sequences from the 44 looked capable of lactam hydrolysis seems to indicate a non-lactam-hydrolysing ancestor. However, this would assume that extant metallo-β-lactamases are a suitable basis for modelling ancestral metallo-β-lactamases, and that our bootstrap sample of phylogenies is unbiased with respect to the true phylogeny. However, neither is likely to be the case. The properties of long-extinct sequences are currently unknowable; if we could ‘rewind the clock’ and replay evolutionary history with its varying selection pressures, we would find that certain evolutionary trajectories are more likely than others, as exemplified in the work of Weinreich et al. (2006). The five trees that do suggest a single origin (representing 5 % of the bootstrap sample) are intriguing, but inconclusive. If—speculatively—they did happen to represent a feasible evolutionary trajectory through structure and sequence space linking a lactam-hydrolysing common ancestor to the extant B1/B2 and B3 enzymes, then Occam’s razor may suggest that this is a likely pathway for evolution to have taken, avoiding as it does the need for a second origin of the same molecular function. Such an interpretation of our results would favour a single evolutionary origin of the metallo-β-lactamase function. This would depend, of course, on the assumption that independent origins of the same function within a superfamily are unlikely. That is a common assumption, based on the perceived difficulty of evolution happening upon the appropriate machinery for catalysing a new chemical reaction unrelated to a protein’s existing function (Babbitt and Gerlt 1997). Nonetheless, we have earlier noted some likely examples of related reactions evolving in the same superfamily (Furnham et al. 2012a, b; Bruns et al. 1997; Burroughs et al. 2006). In our current results, the large majority of reconstructions suggest no metallo-β-lactamase functionality in the MRCA. However, the possibility of ancient lactamases having different structural features from extant ones and the Occam’s razor argument in favour of the one evolutionary trajectory that requires only a single origin of lactam hydrolysis mean that our results lead to an estimate only of a lower bound on the evidence for this functionality, without any clear upper bound. On the basis of our analyses, neither the hypothesis of a single origin nor the hypothesis of two origins can be ruled out.

## Conclusions

To claim to have unequivocally reconstructed the MRCA of the metallo-β-lactamases, let alone to have unambiguously determined its function, would be hubris. Due to uncertainties in phylogeny reconstruction and the lack of any means to parameterise an evolutionary simulation of population-level evolution over billions of years, this is not currently possible and may never be so. Obviously, this in silico study is, unlike real-world evolutionary processes in the long term, not constrained to reconstruct a protein with a biologically useful function. Clearly, we cannot definitively assign function to our ensemble of 98 reconstructed MRCAs. However, the fact that no more than 5 % of the bootstrap sample suggests a lactam-hydrolysing common ancestor supports the contention of Hall and Barlow (Hall et al. 2004) that metallo-β-lactamase activity is most likely to have evolved twice within the same homologous superfamily. If indeed evolution of lactam hydrolysis has occurred twice within this superfamily, the ‘substrate-flexible’ active site is likely to adapt to binding and hydrolysing different lactam derivatives, whereas metal coordination is more constrained and appears less flexible in evolution. However, because of necessary methodological constraints in assessing function and reconstructing ancestors, this 5 % is really a lower bound on evidence for beta-lactamase activity.

Thus, our results do not lend unambiguous support to either hypothesis of one origin or of two separate origins of metallo-β-lactamase function. Rather, it is necessary to assess firstly our ensembles of phylogenetic reconstructions in a way that does not naively assume that importance is proportional to frequency in our bootstrap sample, secondly reliability of functional inferences from matches of homology models to sequence signatures and catalytic templates being mindful that ancient lactamases may have had different active site machinery from modern ones, thirdly the inherent probability of independent inventions of the same function within a homologous superfamily, and fourthly and importantly existing evidence from other studies.

Identifying the functional capabilities of the common ancestor of the metallo-β-lactamases is important in terms of predicting future evolutionary trajectories of these medically significant enzymes, and key in determining the direction of future drug discovery efforts. Particularly given this, it would be conventional—in the face of ambiguous conclusions—to propose a research programme to resolve the ambiguity. However, we see no plausible means to develop sequence or structural signatures for ancestral proteins in the distant past, or to develop correctly parameterised evolutionary models to reconstruct evolutionary history accurately, incorporating interacting fitness effects, selection pressures and population sizes at all times. Engineering our reconstructed ancestral protein and assaying its functionality in the laboratory could be helpful. However, as we have indicated, there are biologically plausible reasons why we might not fully ‘believe’ this reconstruction. We suggest that the question of whether there were one or two origins of metallo-β-lactamase functionality cannot currently be answered with certainty.

## Methods

### FunTree alignment

FunTree generates a structurally informed multiple sequence alignment of the superfamily, which we used as a basis for further analysis (FunTree 3.60.15.10 SSG1) (Furnham et al. 2012a, b). Visual inspection of the alignment revealed that catalytic residues expected to perform similar functional roles, such as metal coordination, were well aligned.

To reduce bias in phylogeny reconstruction due to ‘long branch attraction’ (Felsenstein 1978; Huelsenbeck 1998), additional sequences were added beyond those in the FunTree multiple alignment to break up long branches (Hendy and Penny 1989; Bergsten 2005; Holton and Pisani 2010).

### Additional sequence retrieval from databases

HMMR (Finn et al. 2011) was used to construct a profile Hidden Markov Model (HMM) from the pre-existing FunTree multiple alignment. This FunTree alignment corresponds to a SSG within CATH H-level superfamily 3.60.15.10.

Our profile HMM was used to search the UniProtKB database, using HMMR with default parameters. Additional sequences were added whilst maintaining approximately the same proportion of members in each functional class as the FunTree multiple alignment, since these proportions are biologically meaningful. HMMR hits were ordered by score and chosen by keywords such as ‘flavoprotein’, ‘nitric oxide reductase’ (NOR), ‘Hydroxyglutathionehydrolase’/‘glyoxalase II’ and for the metallo-β-lactamases, we used keywords for different functional members listed by Bebrone (Bebrone 2007) to ensure that a diverse group was picked. Draft sequences were excluded but not all sequences chosen were reviewed. After this, all sequences and structures were checked in Gene3D (Lees et al. 2010, 2012) and CATH, respectively, for the prediction or presence of a 3.60.15.10 domain. Sequences were trimmed at the beginning to remove signal peptide according to PDBsum (Laskowski 2009) or Gene3D. For the flavoproteins/NORs which contain multiple domains, we trimmed both ends of the sequence to extract only the 3.60.15.10 domain as defined by PDBsum for structures and Gene3D for sequences.

### Choice of Outgroup

The metallo-β-lactamase CATH superfamily 3.60.15.10 is composed of two SSGs. Members of SSG2, which mainly consists of ribonucleases (tRNase Z), were used as the outgroup since they form a homologous but structurally distinct group to ingroup members from SSG1. We used sequences of three members of the second SSG that were structurally solved and, hence, had their functional residues designated by experimental means. Only trees in which the ingroup was monophyletic were used in further analysis, therefore reducing the tree set from 100 to 98 members.

### Alignment of additional sequences to pre-existing FunTree alignment

We used the profile aligning facility in MAFFT (Katoh et al. 2005; Katoh and Frith 2012; Katoh and Toh 2008) to align the additional sequences and outgroup using the L-INS-I algorithm, JTT 100 matrix with gap opening and extension penalties of 1.0 and 0.0, respectively. This matrix was chosen based on visual inspection of the alignment, looking for the lowest number of gapped sites and alignment of key catalytic residues.

### Determination of Model and Construction of Phylogenetic trees

A model of protein substitution was selected using Modelgenerator (Keane et al. 2006) with four Gamma categories. BIC, AIC and AIC2 criteria all selected the WAG+I+G (Whelan and Goldman 2001) model. Maximum-likelihood trees were built in PhyML version 3.0 (Guindon et al. 2010) allowing PhyML to optimise the I and G parameters, with the best of ‘Nearest Neighbour Interchange’ and ‘Subtree Pruning and Regrafting’ rearrangements and 100 bootstrap replicates. The ‘Root’ function available for R (The R Development Core Team 2011) using the ‘Ape’ (Paradis et al. 2004) package was used to manually confirm the monophyly of the ingroup in the bootstrap set, resulting in the use of 98 trees for further analysis.

### Reconstruction of 98 ancestral sequences

For the 98 trees, branch lengths were set to a minimum of 0.0001, and sequences were submitted to GASP (Edwards and Shields 2004). GASP was used to reconstruct ancestral sequences for each node in each tree of the bootstrap set, with a WAG substitution matrix, the specified outgroup sequences and other parameters at default settings.

### MRCA node selection

Output trees from ‘GASP’ were viewed in R (The R Development Core Team 2011) using the ‘Ape’ package (Paradis et al. 2004). The node number for the MRCA for each tree was noted and used to extract the relevant GASP predicted sequence for each tree in the bootstrap set using the ‘SeqinR’ package (Charif and Lobry 2007) in R.

### Protein signature searching and clustering of MRCA sequences

The 98 MRCA sequences were submitted to InterProScan. The 44 sequences matching signature IPR001018 were deemed possible ‘metallo-β-lactamases’. These 44 sequences were clustered at 60 % in CD-HIT with default settings, leading to 11 clusters. Representative sequences were designated as by CD-HIT.

### Homology modelling

The 11 representative sequences (one from each cluster) were submitted to Phyre2 (Kelley and Sternberg 2009; Wass et al. 2010). The coordinates of the top homology model, as determined by Phyre2, were used as MRCA models for each cluster.

### Construction of catalytic site templates

PDB structures of B1, B2 and B3 metallo-β-lactamases were chosen if they had records in either MACiE (Holliday et al. 2012) or the Catalytic Site Atlas (CSA) (Porter et al. 2004). Catalytic residues were chosen based on these CSA or MACiE entries.

### Structural alignment of MRCA models and catalytic templates

Structural alignments were performed using CEAlign (Shindyalov and Bourne 1998) in PyMOL Version 1.6.0.0 (Schrodinger LLC 2010), since this is based upon aligning secondary structure rather than primary sequence and is, therefore, more appropriate for aligning evolutionarily divergent proteins.

### Filtering metallo-β-lactamase MRCA candidates

After structural alignment to each of the B1, B2 & B3 catalytic templates, two distance-measurement filters were applied. The first filter was to include an MRCA model as a candidate for possessing lactamase activity if an equivalent residue (identity) could be found within 5.0 angstroms of a catalytic residue of the template. This left us with a range of MRCA structural models with appropriate catalytic residues in a location within a radius not too far away from where we would expect if it were a functional metallo-β-lactamase (based on catalytic residue templates). The next filter was based on the distance *between* catalytic residues. We used the distance found between catalytic residues in the templates as a benchmark figure, and then set a threshold of 2.0 angstroms on either side of this figure. Models which passed the first criterion are, hence, further examined to see whether their putative catalytic residues, selected in the first filter, have a distance between them which would be comparable to that seen in the corresponding metallo-β-lactamase template. The thresholds in both filters, 5.0 and 2.0 angstroms, respectively, are fairly ‘generous’. This is to allow for the fact that homology modelling is of course imprecise, especially since many of the catalytic site residues lie in loop regions. It also allows for the fact that our modelled MRCAs have not been subject to the selective pressure that would be present in nature and might act to constrain the active site (Meng et al. 2004; Torrance et al. 2005).

## Electronic supplementary material

Below is the link to the electronic supplementary material.

Supplementary material 1 (DOCX 15 kb)

Supplementary material 1 (ZIP 1738 kb)
