# Supplementary material for: One origin for metallo-β-lactamase activity, or two? An investigation assessing a diverse set of reconstructed ancestral sequences based on a sample of phylogenetic trees
Source: J Mol Evol. 2014 Sep 4;79(3):117–29. doi: 10.1007/s00239-014-9639-7 (PMC4185109; doi:10.1007/s00239-014-9639-7)
Supplement: Supplementary file 1 — Supplementary material 1 (DOCX 15 kb) [file 239_2014_9639_MOESM1_ESM.docx]

# Online Resources

### 1_GASP

This folder includes the full bootstrap sample of trees in NEWICK format, all phylogenetic trees and sequence files from running the GASP program in NEWICK and FASTA format respectively and the WAG matrix used in the running of the GASP program. Explanatory notes are included.

### 2_MRCA

This folder includes the node numbers corresponding to the MRCA of the metallo-β-lactamases in each of the 98 trees output by GASP and the corresponding ancestral sequence predictions. Explanatory notes are included.

### 3. INTERPROSCAN

This folder contains the results of the InterPro search of the 98 MRCA sequence predictions. Explanatory notes are included.

### 4. CD-HIT

This folder contains the results of clustering of sequences at 60% identity in CD-HIT. Explanatory notes are included.

### 5. PHYRE2

This folder includes all of the 11 MRCA models built in PHYRE2. Also included are the distances of between catalytic residues of aligned MRCA models and templates. Explanatory notes are included.
