# Supplementary material for: One origin for metallo-β-lactamase activity, or two? An investigation assessing a diverse set of reconstructed ancestral sequences based on a sample of phylogenetic trees
Source: J Mol Evol. 2014 Sep 4;79(3):117–29. doi: 10.1007/s00239-014-9639-7 (PMC4185109; doi:10.1007/s00239-014-9639-7)
Supplement: Supplementary file 2 — Supplementary material 1 (ZIP 1738 kb) [file 239_2014_9639_MOESM2_ESM.zip › Online_Rescources_to_submit_18_06_14/2_MRCA/2.1 Ancestral_node_numbers.pdf]

tree_number	MRCA_node_number
1	85
2	87
3	83
4	81
5	85
6	84
7	84
8	82
9	81
10	81
11	77
12	87
13	84
14	82
15	82
16	87
17	84
18	85
19	87
20	84
21	85
22	85
23	84
24	84
25	87
26	85
27	83
28	87
29	84
30	84
31	85
32	82
33	87
34	85
35	87
36	84
37	79
38	85
39	84
40	85
41	84
42	83
43	84
44	72
45	85
46	87
47	84
48	82
49	81

50	84
51	83
52	85
53	82
54	79
55	85
56	85
57	84
58	79
59	82
60	83
61	83
63	85
64	84
65	72
66	84
67	85
68	84
69	81
70	83
71	84
72	84
73	85
74	81
75	84
76	80
77	83
78	83
79	80
80	84
81	85
82	84
83	82
84	85
85	84
86	84
87	87
88	81
89	85
90	82
91	87
92	79
94	84
95	82
96	83
97	81
98	80
99	84
100	84
