# Supplementary material for: One origin for metallo-β-lactamase activity, or two? An investigation assessing a diverse set of reconstructed ancestral sequences based on a sample of phylogenetic trees
Source: J Mol Evol. 2014 Sep 4;79(3):117–29. doi: 10.1007/s00239-014-9639-7 (PMC4185109; doi:10.1007/s00239-014-9639-7)
Supplement: Supplementary file 2 — Supplementary material 1 (ZIP 1738 kb) [file 239_2014_9639_MOESM2_ESM.zip › Online_Rescources_to_submit_18_06_14/3_INTERPROSCAN/InterProScan_results.pdf]

Sequence_number	scanning_service	source_database_signature_accession	source_database_signature_description	match_start	match_end	interpro_accession	interpro_name	gene_ontology_terms
1	PatternScan	PS00743	BETA_LACTAMASE_B_1	68	88	IPR001018	Beta-lactamase, class-B, conserved site	Molecular Function: zinc ion binding (GO:0008270), Molecular Function: beta-lactamase activity (GO:0008800), Biological Process: antibiotic catabolic process (GO:0017001)
2	PatternScan	PS00743	BETA_LACTAMASE_B_1	67	87	IPR001018	Beta-lactamase, class-B, conserved site	Molecular Function: zinc ion binding (GO:0008270), Molecular Function: beta-lactamase activity (GO:0008800), Biological Process: antibiotic catabolic process (GO:0017001)
4	PatternScan	PS00743	BETA_LACTAMASE_B_1	62	82	IPR001018	Beta-lactamase, class-B, conserved site	Molecular Function: zinc ion binding (GO:0008270), Molecular Function: beta-lactamase activity (GO:0008800), Biological Process: antibiotic catabolic process (GO:0017001)
5	PatternScan	PS00743	BETA_LACTAMASE_B_1	70	90	IPR001018	Beta-lactamase, class-B, conserved site	Molecular Function: zinc ion binding (GO:0008270), Molecular Function: beta-lactamase activity (GO:0008800), Biological Process: antibiotic catabolic process (GO:0017001)
6	PatternScan	PS00743	BETA_LACTAMASE_B_1	55	75	IPR001018	Beta-lactamase, class-B, conserved site	Molecular Function: zinc ion binding (GO:0008270), Molecular Function: beta-lactamase activity (GO:0008800), Biological Process: antibiotic catabolic process (GO:0017001)
7	PatternScan	PS00743	BETA_LACTAMASE_B_1	68	88	IPR001018	Beta-lactamase, class-B, conserved site	Molecular Function: zinc ion binding (GO:0008270), Molecular Function: beta-lactamase activity (GO:0008800), Biological Process: antibiotic catabolic process (GO:0017001)
8	PatternScan	PS00743	BETA_LACTAMASE_B_1	66	86	IPR001018	Beta-lactamase, class-B, conserved site	Molecular Function: zinc ion binding (GO:0008270), Molecular Function: beta-lactamase activity (GO:0008800), Biological Process: antibiotic catabolic process (GO:0017001)
10	PatternScan	PS00743	BETA_LACTAMASE_B_1	68	88	IPR001018	Beta-lactamase, class-B, conserved site	Molecular Function: zinc ion binding (GO:0008270), Molecular Function: beta-lactamase activity (GO:0008800), Biological Process: antibiotic catabolic process (GO:0017001)
13	PatternScan	PS00743	BETA_LACTAMASE_B_1	67	87	IPR001018	Beta-lactamase, class-B, conserved site	Molecular Function: zinc ion binding (GO:0008270), Molecular Function: beta-lactamase activity (GO:0008800), Biological Process: antibiotic catabolic process (GO:0017001)
14	PatternScan	PS00743	BETA_LACTAMASE_B_1	71	91	IPR001018	Beta-lactamase, class-B, conserved site	Molecular Function: zinc ion binding (GO:0008270), Molecular Function: beta-lactamase activity (GO:0008800), Biological Process: antibiotic catabolic process (GO:0017001)
16	PatternScan	PS00743	BETA_LACTAMASE_B_1	67	87	IPR001018	Beta-lactamase, class-B, conserved site	Molecular Function: zinc ion binding (GO:0008270), Molecular Function: beta-lactamase activity (GO:0008800), Biological Process: antibiotic catabolic process (GO:0017001)
17	PatternScan	PS00743	BETA_LACTAMASE_B_1	62	82	IPR001018	Beta-lactamase, class-B, conserved site	Molecular Function: zinc ion binding (GO:0008270), Molecular Function: beta-lactamase activity (GO:0008800), Biological Process: antibiotic catabolic process (GO:0017001)
18	PatternScan	PS00743	BETA_LACTAMASE_B_1	65	85	IPR001018	Beta-lactamase, class-B, conserved site	Molecular Function: zinc ion binding (GO:0008270), Molecular Function: beta-lactamase activity (GO:0008800), Biological Process: antibiotic catabolic process (GO:0017001)
19	PatternScan	PS00743	BETA_LACTAMASE_B_1	68	87	IPR001018	Beta-lactamase, class-B, conserved site	Molecular Function: zinc ion binding (GO:0008270), Molecular Function: beta-lactamase activity (GO:0008800), Biological Process: antibiotic catabolic process (GO:0017001)
19	PatternScan	PS00744	BETA_LACTAMASE_B_2	144	156	IPR001018	Beta-lactamase, class-B, conserved site	Molecular Function: zinc ion binding (GO:0008270), Molecular Function: beta-lactamase activity (GO:0008800), Biological Process: antibiotic catabolic process (GO:0017001)
20	PatternScan	PS00743	BETA_LACTAMASE_B_1	57	77	IPR001018	Beta-lactamase, class-B, conserved site	Molecular Function: zinc ion binding (GO:0008270), Molecular Function: beta-lactamase activity (GO:0008800), Biological Process: antibiotic catabolic process (GO:0017001)
21	PatternScan	PS00743	BETA_LACTAMASE_B_1	63	83	IPR001018	Beta-lactamase, class-B, conserved site	Molecular Function: zinc ion binding (GO:0008270), Molecular Function: beta-lactamase activity (GO:0008800), Biological Process: antibiotic catabolic process (GO:0017001)
22	PatternScan	PS00743	BETA_LACTAMASE_B_1	56	76	IPR001018	Beta-lactamase, class-B, conserved site	Molecular Function: zinc ion binding (GO:0008270), Molecular Function: beta-lactamase activity (GO:0008800), Biological Process: antibiotic catabolic process (GO:0017001)
24	PatternScan	PS00743	BETA_LACTAMASE_B_1	57	77	IPR001018	Beta-lactamase, class-B, conserved site	Molecular Function: zinc ion binding (GO:0008270), Molecular Function: beta-lactamase activity (GO:0008800), Biological Process: antibiotic catabolic process (GO:0017001)
25	PatternScan	PS00743	BETA_LACTAMASE_B_1	58	78	IPR001018	Beta-lactamase, class-B, conserved site	Molecular Function: zinc ion binding (GO:0008270), Molecular Function: beta-lactamase activity (GO:0008800), Biological Process: antibiotic catabolic process (GO:0017001)
26	PatternScan	PS00743	BETA_LACTAMASE_B_1	59	79	IPR001018	Beta-lactamase, class-B, conserved site	Molecular Function: zinc ion binding (GO:0008270), Molecular Function: beta-lactamase activity (GO:0008800), Biological Process: antibiotic catabolic process (GO:0017001)
28	PatternScan	PS00743	BETA_LACTAMASE_B_1	66	86	IPR001018	Beta-lactamase, class-B, conserved site	Molecular Function: zinc ion binding (GO:0008270), Molecular Function: beta-lactamase activity (GO:0008800), Biological Process: antibiotic catabolic process (GO:0017001)
34	PatternScan	PS00743	BETA_LACTAMASE_B_1	67	87	IPR001018	Beta-lactamase, class-B, conserved site	Molecular Function: zinc ion binding (GO:0008270), Molecular Function: beta-lactamase activity (GO:0008800), Biological Process: antibiotic catabolic process (GO:0017001)
35	PatternScan	PS00743	BETA_LACTAMASE_B_1	64	84	IPR001018	Beta-lactamase, class-B, conserved site	Molecular Function: zinc ion binding (GO:0008270), Molecular Function: beta-lactamase activity (GO:0008800), Biological Process: antibiotic catabolic process (GO:0017001)
41	PatternScan	PS00743	BETA_LACTAMASE_B_1	55	75	IPR001018	Beta-lactamase, class-B, conserved site	Molecular Function: zinc ion binding (GO:0008270), Molecular Function: beta-lactamase activity (GO:0008800), Biological Process: antibiotic catabolic process (GO:0017001)
46	PatternScan	PS00744	BETA_LACTAMASE_B_2	144	156	IPR001018	Beta-lactamase, class-B, conserved site	Molecular Function: zinc ion binding (GO:0008270), Molecular Function: beta-lactamase activity (GO:0008800), Biological Process: antibiotic catabolic process (GO:0017001)
51	PatternScan	PS00743	BETA_LACTAMASE_B_1	72	92	IPR001018	Beta-lactamase, class-B, conserved site	Molecular Function: zinc ion binding (GO:0008270), Molecular Function: beta-lactamase activity (GO:0008800), Biological Process: antibiotic catabolic process (GO:0017001)
56	PatternScan	PS00743	BETA_LACTAMASE_B_1	54	74	IPR001018	Beta-lactamase, class-B, conserved site	Molecular Function: zinc ion binding (GO:0008270), Molecular Function: beta-lactamase activity (GO:0008800), Biological Process: antibiotic catabolic process (GO:0017001)
60	PatternScan	PS00743	BETA_LACTAMASE_B_1	65	85	IPR001018	Beta-lactamase, class-B, conserved site	Molecular Function: zinc ion binding (GO:0008270), Molecular Function: beta-lactamase activity (GO:0008800), Biological Process: antibiotic catabolic process (GO:0017001)
61	PatternScan	PS00743	BETA_LACTAMASE_B_1	78	98	IPR001018	Beta-lactamase, class-B, conserved site	Molecular Function: zinc ion binding (GO:0008270), Molecular Function: beta-lactamase activity (GO:0008800), Biological Process: antibiotic catabolic process (GO:0017001)
63	PatternScan	PS00743	BETA_LACTAMASE_B_1	61	81	IPR001018	Beta-lactamase, class-B, conserved site	Molecular Function: zinc ion binding (GO:0008270), Molecular Function: beta-lactamase activity (GO:0008800), Biological Process: antibiotic catabolic process (GO:0017001)
66	PatternScan	PS00743	BETA_LACTAMASE_B_1	77	97	IPR001018	Beta-lactamase, class-B, conserved site	Molecular Function: zinc ion binding (GO:0008270), Molecular Function: beta-lactamase activity (GO:0008800), Biological Process: antibiotic catabolic process (GO:0017001)
70	PatternScan	PS00743	BETA_LACTAMASE_B_1	65	85	IPR001018	Beta-lactamase, class-B, conserved site	Molecular Function: zinc ion binding (GO:0008270), Molecular Function: beta-lactamase activity (GO:0008800), Biological Process: antibiotic catabolic process (GO:0017001)
71	PatternScan	PS00743	BETA_LACTAMASE_B_1	68	88	IPR001018	Beta-lactamase, class-B, conserved site	Molecular Function: zinc ion binding (GO:0008270), Molecular Function: beta-lactamase activity (GO:0008800), Biological Process: antibiotic catabolic process (GO:0017001)
72	PatternScan	PS00744	BETA_LACTAMASE_B_2	132	144	IPR001018	Beta-lactamase, class-B, conserved site	Molecular Function: zinc ion binding (GO:0008270), Molecular Function: beta-lactamase activity (GO:0008800), Biological Process: antibiotic catabolic process (GO:0017001)
74	PatternScan	PS00743	BETA_LACTAMASE_B_1	54	74	IPR001018	Beta-lactamase, class-B, conserved site	Molecular Function: zinc ion binding (GO:0008270), Molecular Function: beta-lactamase activity (GO:0008800), Biological Process: antibiotic catabolic process (GO:0017001)
80	PatternScan	PS00743	BETA_LACTAMASE_B_1	58	78	IPR001018	Beta-lactamase, class-B, conserved site	Molecular Function: zinc ion binding (GO:0008270), Molecular Function: beta-lactamase activity (GO:0008800), Biological Process: antibiotic catabolic process (GO:0017001)
82	PatternScan	PS00743	BETA_LACTAMASE_B_1	71	91	IPR001018	Beta-lactamase, class-B, conserved site	Molecular Function: zinc ion binding (GO:0008270), Molecular Function: beta-lactamase activity (GO:0008800), Biological Process: antibiotic catabolic process (GO:0017001)
83	PatternScan	PS00743	BETA_LACTAMASE_B_1	64	84	IPR001018	Beta-lactamase, class-B, conserved site	Molecular Function: zinc ion binding (GO:0008270), Molecular Function: beta-lactamase activity (GO:0008800), Biological Process: antibiotic catabolic process (GO:0017001)
86	PatternScan	PS00743	BETA_LACTAMASE_B_1	64	84	IPR001018	Beta-lactamase, class-B, conserved site	Molecular Function: zinc ion binding (GO:0008270), Molecular Function: beta-lactamase activity (GO:0008800), Biological Process: antibiotic catabolic process (GO:0017001)
87	PatternScan	PS00743	BETA_LACTAMASE_B_1	54	74	IPR001018	Beta-lactamase, class-B, conserved site	Molecular Function: zinc ion binding (GO:0008270), Molecular Function: beta-lactamase activity (GO:0008800), Biological Process: antibiotic catabolic process (GO:0017001)
88	PatternScan	PS00743	BETA_LACTAMASE_B_1	69	89	IPR001018	Beta-lactamase, class-B, conserved site	Molecular Function: zinc ion binding (GO:0008270), Molecular Function: beta-lactamase activity (GO:0008800), Biological Process: antibiotic catabolic process (GO:0017001)
90	PatternScan	PS00743	BETA_LACTAMASE_B_1	69	89	IPR001018	Beta-lactamase, class-B, conserved site	Molecular Function: zinc ion binding (GO:0008270), Molecular Function: beta-lactamase activity (GO:0008800), Biological Process: antibiotic catabolic process (GO:0017001)
94	PatternScan	PS00743	BETA_LACTAMASE_B_1	57	77	IPR001018	Beta-lactamase, class-B, conserved site	Molecular Function: zinc ion binding (GO:0008270), Molecular Function: beta-lactamase activity (GO:0008800), Biological Process: antibiotic catabolic process (GO:0017001)
95	PatternScan	PS00743	BETA_LACTAMASE_B_1	79	99	IPR001018	Beta-lactamase, class-B, conserved site	Molecular Function: zinc ion binding (GO:0008270), Molecular Function: beta-lactamase activity (GO:0008800), Biological Process: antibiotic catabolic process (GO:0017001)
